# Biological and molecular characterization of a sheep pathogen isolate of *Mannheimia haemolytica* and leukotoxin production kinetics

**DOI:** 10.14202/vetworld.2021.2031-2040

**Published:** 2021-08-07

**Authors:** Dounia Bkiri, Noha Semmate, Zineb Boumart, Najete Safini, Fatima Zohra Fakri, Zahra Bamouh, Khalid Omari Tadlaoui, Siham Fellahi, Noursaid Tligui, Ouafaa Fassi Fihri, Mehdi El Harrak

**Affiliations:** 1 Department of Research and Development, Multi-chemical Industry, Mohammedia, Morocco; 2 Department of Microbiology, Immunology and Contagious Diseases, Institute of Agronomy and Veterinary Medicine Hassan II, Rabat, Morocco

**Keywords:** histopathology, isolation, leukotoxin, *Mannheimia haemolytica*, phylogeny, polymerase chain reaction

## Abstract

**Background and Aim:**

*Mannheimia haemolytica* (Mha) is a common agent of pneumonia in ruminants globally, causing economic losses by morbidity, mortality, and treatment costs. Infection by Mha is often associated with or promoted by respiratory viral pathogens and environmental conditions. Infections due to Mha have rarely been described in small ruminants. This study reports the biological and molecular characteristics of a new Moroccan Mha isolate from small ruminants presenting typical respiratory symptoms. We also studied the cultural parameters, growth kinetics, and Lkt excretion of the isolate and its pathogenicity on laboratory animals and small ruminants.

**Materials and Methods:**

Suspected pasteurellosis cases in sheep and goat flocks in Morocco were investigated. A local strain of Mha was isolated and identified using biochemical and molecular methods. Polymerase chain reaction-targeting specific genes were used for serotyping and phylogenetic analyses; further, leukotoxin production, cytotoxicity, and pathogenicity of the isolate in mice, goats, and sheep were investigated.

**Results:**

Phylogeny analysis revealed 98.76% sequence identity with the USA isolate of 2013; the strain growth with a cycle of 9-10 h with leukotoxin secretion was detected by NETosis and quantified by cytotoxicity and mortality of mice. Goat and sheep infections cause hyperthermia, with characteristic postmortem lesions in the trachea and lung.

**Conclusion:**

A local isolate of Mha from sheep that died of pneumonia was characterized for the 1^st^ time in North Africa using biological and molecular methods. Although growth on appropriate culture media is accompanied by intense leukotoxin secretion, experimental infections of sheep and goats cause hyperthermia and typical lesions of pneumonia.

## Introduction

Mannheimiosis is a common respiratory tract infection that is highly prevalent in ruminants [1]. It is caused by *Mannheimia haemolytica* (Mha), a Gram-negative, optional anaerobic, nonmotile, and opportunistic pathogen. This organism changes from a benign commensal to a deadly pathogen by colonizing the lower respiratory tract during stress (viral infection, physical condition, transport, immuno-depression, and environment), causing variable pneumonia types [2]. Clinical signs vary in intensity from frustrating to severe, rapidly fatal infection, characterized by anorexia, tachycardia, weight loss, rhinitis, cough, and polypnea, followed by dyspnea. Mha infection causes significant economic losses in the production chain due to severe morbidity and mortality [3].

Twelve A serotypes were identified (A1, A2, A5, A6, A7, A8, A9, A12, A13, A14, A16, and A17) as Mha, whereas the remaining A11 serotypes became *Mannheimia glucosida* [4,5]. Among these serotypes, A1 and A2 are the most common globally, but their prevalence may vary by region; however, A1 is known as the major causative agent of mannheimiosis. The bacterium produces several virulence factors, the most important of which is the leukotoxin (Lkt) secreted during the exponential growth phase [6]. Lkts are a group of exotoxins that produce primary toxic effects on leukocytes [7]. This exotoxin (Lkt) causes pulmonary alveolar damage from enzymes and oxygen free radicals released from Lkt-damaged leukocytes, resulting in cell bursting and degranulation, releasing an inflammatory cytokine at high concentration, and causing necrosis and degradation of the lung’s immune system [7,8]. Lkt is a protein characterized by an approximate molecular weight of 102 kDa. The toxic component of Lkt is located in the N-terminus, whereas the region stimulating Lkt-neutralizing antibodies is localized in the 32-amino-acid region near the C-terminus [9]. Thus, the toxin is specific for leukocytes from ruminant species. Lkt is excreted into the culture supernatant, especially in the logarithmic phase, binds to the Lkt receptor β2 integrin, CD18, and induces dose-related changes in ruminant leukocytes [10]. Mannheimiosis is cosmopolitan; however, data on the epidemiology of pneumonia associated with Mha are limited. To date, few studies have been published on the isolation and identification of Mha in Morocco. Recent works have reported the characterization and isolation of Mha Morocco strains [11]. Efficacious Mha vaccines could potentially reduce the severity of or prevent pneumonia in ruminants.

This study reports the biological and molecular characteristics of a new Moroccan Mha isolate from small ruminants presenting typical respiratory symptoms. We also studied the cultural parameters, growth kinetics, and Lkt excretion of the isolate and its pathogenicity on laboratory animals and small ruminants.

## Materials and Methods

### Ethical approval

Experiments on mice, sheep, and goats were carried out according to International Guidelines described for the care and handling of experimental animals, chapter 7.8 of the Terrestrial Animal Health Code and Directive 2010/63/UE of the European Commission. The protocol was submitted and approved by the Institutional Ethics Committee for Animal Experiments.

### Study period and location

The study was conducted from January 2019 to December 2020 at Department of Research and Development, Multi-chemical Industry, Mohammedia, Morocco.

### Case description

Clinical symptoms of pneumonia with mortality are observed regularly in cattle, sheep, and goats in a flock in Morocco. During the winter of 2019, in a flock of around 300 sheep in the northwest of Morocco (Sidi Yahya Zaers), ten animals presented hyperthermia, anorexia, cough, dyspnea, and mucopurulent discharge. Postmortem examinations revealed pneumonia lesions and pleurisy with fibrinous pleural effusion in the animals. Lungs were edematous and hemorrhagic, with exudative mucopurulent inflammation. A specimen from the lung of a dead newborn lamb was sampled for this study.

### Sample preparation and isolation

A 24-h pre-enrichment culture at 37°C was prepared using tryptone soy broth media. The suspension was inoculated into an agar-based medium supplemented with 5% sheep blood and incubated at 37°C for 24 h under aerobic conditions.

Typical white–gray, round, medium-sized, and non-mucoid colonies with hemolysis were subjected to Gram staining, revealing Gram-negative coccobacillus. Primary identification was made by biochemical testing using commercially available tests (catalase, oxidase, and API gallery API 20NE). Positive catalase and oxidase colonies subsequently identified Mha by polymerase chain reaction (PCR) as described below. The isolate was stored at −80°C with 20% glycerol until use [12].

### Molecular identification and genotyping

DNA was extracted from 200 μL of fresh bacterial culture using the Isolate II Genomic DNA Kit(Bioline, USA) and eluted in 100 μL of elution buffer, according to the manufacturer’s instructions. PCR assays targeting methyltransferase (mod) and restriction endonuclease (res) genes were used for the molecular detection of Mha [13]. The reaction was performed in 96-well Optical Reaction Plates (Applied Biosystems, USA), containing 1× PCR buffer (Invitrogen, USA), 1.5 mM MgCl_2_, 0.2 mM dNTP, 0.4 mM of each primer, two units of Taq polymerase, and 1 μg of the DNA template, with a final volume of 25 μL. Reactions were run on the GeneAmp PCR System 9700 using the following amplification programs: initial denaturation at 95°C for 3 min, 30 cycles of denaturation at 95°C for 1 min, annealing at 55°C for 1 min, and extension at 72°C for 30 s. The PCR products obtained were resolved by electrophoresis on a 1.5% (w/v) agarose gel 1-kb DNA marker ladder (Biolabs, USA). Gels were visualized after staining with ethidium bromide.

Serotyping was performed using PCR assays by the amplification of hypothetical protein gene forward (5′-CAT TTC CTT AGG TTC AGC-3′) and reverse (5′-CAA GTC ATC GTA ATG CCT-3′) primers for serotype 1-specific detection [14].

### PCR for *Rpt2* gene detection and DNA sequencing

Amplification of the *Rpt2* gene sequence by PCR was performed using the *Rpt2* forward (5′-GTT TGT AAG ATA TCC CAT TT-3′) and reverse (5′-CGT TTT CCA CTT GCG TGA-3′) primers [15]. PCR was performed using the Applied Biosystems kit protocol, in a 20- μL reaction mixture containing 2 μL buffer (10×), 2.5 μL MgCl_2 (_25 mmol/l), 2.5 μL dNTP (10 mmol/l), 0.75 μL of each primer (10 μmol/l), 10.2 μL of sterilized water, 0.5 μL of RNase inhibitor (20 U/μL), 0.3 μL RT (50 U/μL), and 0.5 μL of Gold Taq polymerase (5 U/μL). PCR products were analyzed on a 1% agarose gel, and the *Rpt2* gene (1000 bp) was purified using the Gene Clean Kit (ExoSAP, USA). Purified PCR products were used as templates for sequencing using the BigDye Terminator v.1.1 Cycle Sequencing Kit. The second purification step was performed using the BigDye Terminator Purification Kit (Life Technologies, USA). Purified PCR products were sequenced in both directions using the same primers.

### Sequence and phylogenetic analyses

*Rpt2* sequence data were assembled manually using BioEdit software package v.5.0.9 (https://bioedit.software.informer.com/5.0/) [16]. The open-source BLAST program (http://blast.ncbi.nlm.nih.gov/Blast.cgi) was used for sequence comparison. A phylogenetic tree was generated using the maximum likelihood method, a general time-reversible model, with 500 bootstrap replicates with MEGA 6.06 (https://www.megasoftware.net/show_eua), and bootstrap values above 50 were labeled on major tree branches. Finally, the sequences were submitted to the EMBL/GenBank databases [17].

### Growth kinetics

Mha was first grown on brain heart infusion (BHI) agar for 9 h at 37°C, then passed in BHI broth and incubated for 12 h at 37°C with moderate agitation. Culture in bioreactor was conducted at 35°C with 100 rpm agitation for 10 h [6]. At the determined optical density (OD) values, culture samples were taken every hour for leukotoxin and bacterial titration and pathogenicity studies in mice. In addition, growth kinetics was evaluated.

### Protein electrophoresis and Western blotting of the leukotoxin

For the specific detection of Mha leukotoxin and its expression level, Western blotting was performed. Supernatant proteins were resolved by sodium dodecyl sulfate-polyacrylamide gel electrophoresis (SDS-PAGE) using 10% running gel (Tris–HCl buffer, pH 8.8). Electrophoresis was conducted in a Tris-glycine chamber buffer at two-step 100 mA for 20 min in the first part of the concentration gel compared with 60 min using 150 mA to separate all existing proteins ideally. The Color Prestained Protein Standard, Broad Range of 11-245 kDa was used as the molecular weight standard. The protein migration obtained was transferred to a 0.45-μm nitrocellulose membrane (Bio-Rad, USA) using an immunoblot system at a constant current of 100 mA for 60 min. Diluted 1/4000 lktA antibody CSB-PA314743EA01ESE (Cusabio, China) was added and incubated for 1 h at room temperature (25°C). First, antibodies were detected by adding a goat anti-rabbit horseradish peroxidase-conjugated antibody. Membranes were then exposed to luminol and hydrogen peroxide using an enhanced chemiluminescence kit (Amershem™ ECL™, USA Western Blotting Detection Reagents) and revealed with a photographic film (GE HealthCare Limited, Amersham Hyperfilm™ ECL, USA).

### Leukotoxin detection by extracellular DNA

Blood from healthy sheep was centrifuged at 1000g for 15 min to remove the plasma and buffy coat. Red blood cells were lysed (150 mM ammonium chloride, 10 mM Tris; pH 7.5). Ovine neutrophils, polymorphonuclear leukocytes (PMNs) were pelleted at 1000 g and washed. Cells were suspended in RPMI medium with 10% fetal bovine serum and examined under a microscope. Cells with a purity of more than 98% PMNs and more than 99% viability, determined by fluorescein diacetate and propidium iodide (FDA/PI) staining using fluorescence microscopy, were deemed acceptable for subsequent use. PMNs (10^6^) were incubated for 2.5 h with the Mha supernatant. Then, 100 mL of the cells was fixed and stained with May-Grunwald–Giemsa, and the rest of the suspension was tested for extracellular DNA quantification. Cells incubated in the presence of the leukotoxin were centrifuged, and the supernatants were removed to add fresh medium and isolate NETs. The mixture was centrifuged at 1000 g for 5 min. Neutrophil extracellular DNA was quantified using Hoechst 33342 (AAT Bioquest, USA) solution at 5 μg/mL. Fluorescence was measured using a plate reader [18].

### Leukotoxin detection by staining

Lkt was detected by inoculating sheep lymphocyte cells with the culture supernatant filtrate as described previously [19]. FDA was absorbed by lymphocyte cells, converting the FDA into a fluorescent green metabolite. The green signal serves as an indicator of viable cells, as the conversion is esterase-dependent. In contrast, the nuclei staining dye, PI, cannot pass through the viable cell membrane; however, it can reach the nucleus by passing through disordered areas of dead cells to intercalate with the cell DNA double helix. Thus, live cells will be fluorescent green and dead cells, fluorescent red [20]. May-Grunewald–Giemsa staining was used to visualize cytopathic effects in neutrophils [21].

### Leukotoxin titration

Sheep blood was sampled on heparin tubes. Phosphate-buffered saline (PBS) was used for blood dilution and lymphocyte washing. The lymphocyte separation medium (WISENT, 305-010-CL) was used for lymphocyte isolation. RPMI medium was used for the final lymphocyte dilution and trypan blue dye solution for cell counting.

The blood was diluted in PBS and then poured carefully over the medium. The separation process was performed by centrifugation at 1200 g for 20 min. Lymphocytes were then recovered by layer aspiration and washed 3 times with PBS, after which the lymphocyte pellet was reconstituted in the medium. A cell suspension was prepared at a final concentration of 5×10^6^ cells/mL in the medium. A volume of 500 μL of culture supernatant was introduced into a vacutainer tube containing 500 μL of lymphocyte suspension, mixed smoothly, and incubated for 2 h in a water bath at 37°C. Afterward, cell counting was performed to evaluate cell viability. A leukotoxicity evaluation was made according to the percentage of killed cells to total cells. A negative control enabled the validation of the test [19].

### Pathogenicity in mice

The pathogenicity and toxicity of the Mha isolate were determined in BALB/c mice, weighing 18-22 g. Seventy mice were divided into three groups: Group 1 (G1; n=30) was inoculated with 0.5 mL of the pure fresh bacterial suspension (1×10^9^ CFU/mL), with 5×10^8^ and 1×10^8^ CFU/mL; and Group 2 (G2; n=30) was inoculated with the supernatant after centrifugation (3000 rpm, 20 min) and filtration (0.2 μm). The filtrate was inoculated intravenously at 0.5 mL per mouse, at different doses, 1×10^9^, 5×10^8^, and 1×10^8^ CFU/mL. One group of mice (n=10) was the control group and injected only with a fresh medium. Animals were observed for 5 day, and their symptoms and mortality were recorded. Dead mice were autopsied, and lung and trachea tissues were sampled for histological studies.

### Pathogenicity in sheep and goats

**Figure-1:**
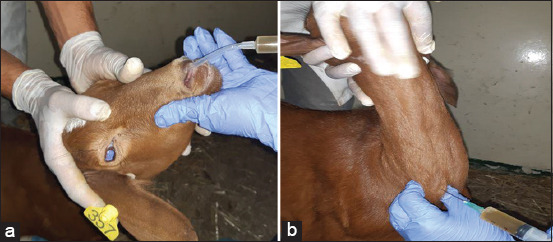
Experimental infection of goats with *Mannheimia haemolytica* by intranasal (a) and intra-tracheal (b) route.

Experimental infection was performed on three healthy male goats and two lambs; three goats were used as controls. The animals were of a local breed and aged 5 months old, weighing an average of 20 kg. Animals were inoculated with a Mha isolate suspension containing 10^9^ bacteria/mL. They were infected atday 0 (D0) and D1 with 2 mL of the suspension in each nostril and 5 mL in the distal part of the trachea using a sterile syringe (Figures-1a and b). Animals were monitored daily for general conditions, rectal temperature, respiratory frequency, nasal secretion, and cough. Moreover, blood samples were taken at D0, D7, and D14 post-infection; nasal and pharyngeal swabs were sampled every 3 days at D0, D3, D6, D9, and D12 post-infection for PCR analyses. Postmortem samples were collected from the trachea, lungs, and pulmonary lymph nodes.

### Necropsy and histopathology

Necropsy was performed on dead and euthanized goats and sheep. Tissue specimens were collected and fixed in 10% neutral formalin and processed for histopathological evaluations. Paraffin tissue sections were cut at 5 μm thickness and stained with hematoxylin and eosin (H&E).

### Serology

Mha-specific antibodies were assessed using a commercial Monoscreen Ab indirect ELISA kit (Bio X Diagnostics, Belgium).

## Results

### Isolation and biochemical identification

Bacterialisolation was performed from the lung tissue of a dead sheep after treatment. From culture-positive plates, typical colonies were subjected to Gram staining for cellular morphology. Gram-negative bacteria were further subcultured on blood and MacConkey agar plates for final identification.

The typical growth colonies were characterized on blood agar plates for the presence and type of hemolysis, morphology, color, shape, size, and consistency, and on MacConkey agar for lactose fermentation. Pure cultures from a single colony were further analyzed by catalase and oxidase tests. The final identification of bacterial species was performed using the biochemical identification kit (API 20NE, bioMerieux, France), according to the supplier’s instructions.

From 11 specimens identified as Mha, only one isolation was performed based on Lkt production. This strain was recovered from tissue lung samples of one sheep. Furthermore, the isolate with positive reactions for catalase and oxidase was confirmed as Mha1 using API 20NE and PCR.

### Molecular detection and serotyping

**Figure-2:**
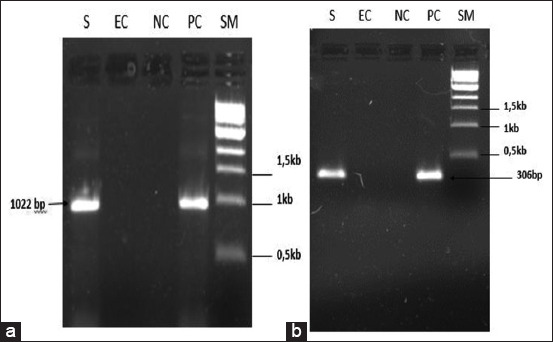
Polymerase chain reaction amplification result of identity (a) and serotyping (b) of *Mannheimia haemolytica* isolate. SM: Size marker 1Kb DNA Ladder; BioLabs; PC: positive control (*M. haemolytica* ATCC^®^ 43270™) NC: Negative control; EC: Extraction control; S: *M. haemolytica* isolated strain.

The isolated strain was identified using PCR as Mha and typed as serotype A1 (Figure-2).

### Phylogenetic analysis

**Figure-3:**
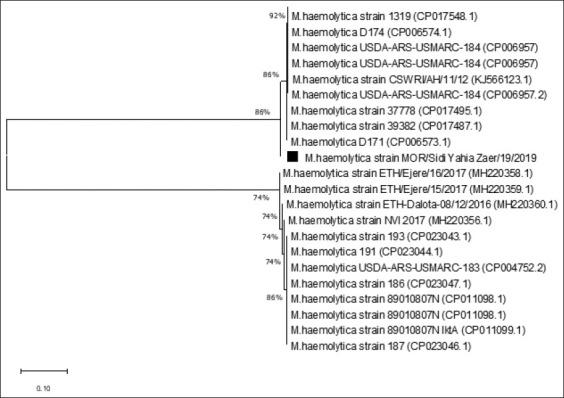
Phylogenetic analysis of *Mannheimia haemolytica* strains using Rpt2 gene sequence.

A phylogenetic tree was constructed from the deduced amino-acid sequences of the *Rpt2* gene of the Mha strain of the Moroccan isolate and Mha-referenced strains. The Mha Moroccan isolate was clustered with an American genotype and formed a common branch (Figure-3). Furthermore, the deduced amino-acid sequences of these Mha isolates were exposed to BLAST and compared with the reference and variant strain sequences retrieved from GenBank from different world regions. The sequence of the Moroccan Mha isolated in this study showed 98.76% amino-acid identity with Mha (USDA/ARS/USMARC/184) isolated in the USA in 2013.

### Growth kinetics

**Figure-4:**
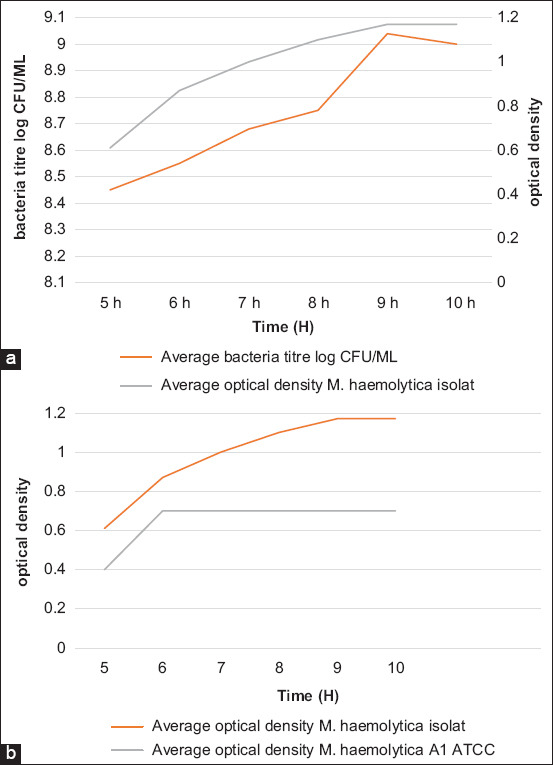
Growth kinetic of *Mannheimia haemolytica* isolate (a) at 37°C in bioreactor (average of 3 essays) and comparison with the reference ATCC strain (b).

The isolated Mha growth kinetics was studied comparatively with the reference strain (Figures-4a and b). During culture on a bioreactor, parameters such as agitation, temperature, OD, and pH were monitored. Samples from the culture were taken every hour and analyzed to evaluate the bacterial concentration and Lkt secretion. The exponential phase was reached after 7-9 h of fermentation, and the culture was stopped after 10 h during the stationary phase. It was noticed that the exponential phase of the culture of the Mha isolate was reached more rapidly than the reference ATCC 43270.

### Protein electrophoresis and Western blotting for leukotoxin detection

**Figure-5:**
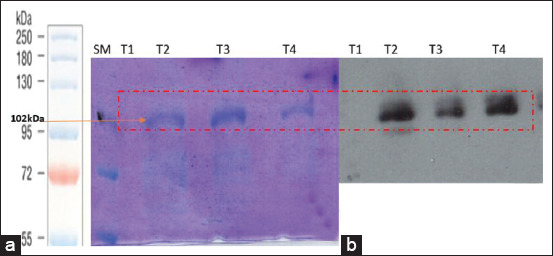
*Mannheimia haemolytica* (Mha) culture growth and Lkt secretion profile. (a): SDS page of Mha culture supernatant samples. (b): Anti-Lkt Western blot of Mha culture supernatant samples. SM: Molecular weight marker (color protein standard broad range P7712S Biolabs).

The supernatant of the isolate was analyzed using SDS-PAGE and specifically identified using an anti-LktA antibody. We obtained a specific band at approximately 102 kDa, detectable by SDS-PAGE and immunological detection confirmed by Western blotting to show the correlation between the protein profile and immunological detection (Figures-5a and b). Lkt secretion was detected after 7 h of culture and reached a peak at 8-9 h.

### Leukotoxin detection by extracellular DNA

**Figure-6:**
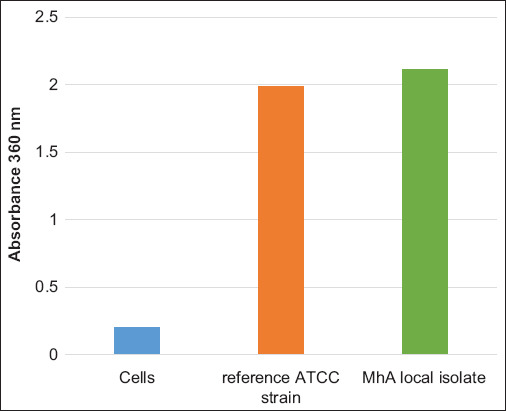
Supernatant containing the lkt components of *Mannheimia haemolytica* local isolate and comparison with the reference ATCC strain was assayed for the presence of extracellular DNA.

Figure-6 demonstrates absorbance values, and there was a significant increase in DNA for NET production between untreated and treated cells; however, we did not notice any significant difference between ATCC 43270 and the Mha isolate treatment.

### Leukotoxin detection by staining

**Figure-7:**
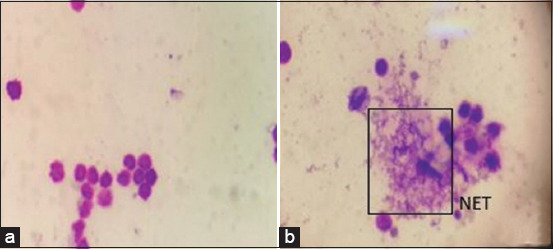
May-Grunwald-Giemsa: negative control (a) and Lkt treated (b) (1000×).

**Figure-8:**
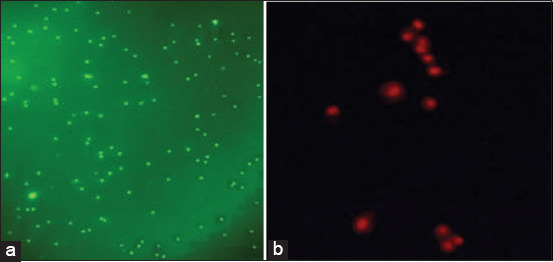
The bright stained green regions are related to the FDA-stained (live) cells (a) The small bright spots correspond to the intense red staining of PI-positive cells (dead) (b) (400×).

Mha Lkt induces NET formation in ovine neutrophils (Figure-7b) compared with untreated PMNs (Figure-7a). NET is the extracellular DNA and associated proteins released by stimulated neutrophils. The leukotoxin (Lkt) of Mha was previously demonstrated to cause NET formation of ruminants’ neutrophils [22]. Cell viability assessments by FDA/PI staining are reported in Figures-8a and b. The FDA/PI method allows us to assess live cells versus dead cells [20].

### Leukotoxin titration

**Figure-9:**
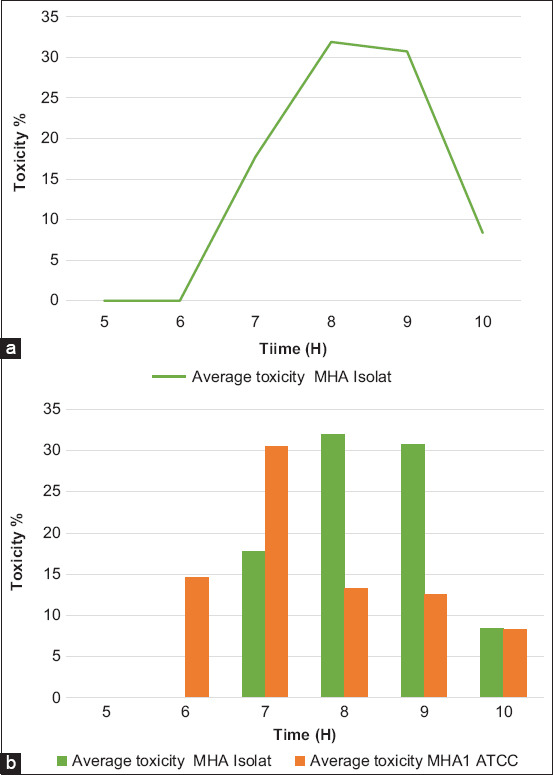
Leukotoxin secretion by *Mannheimia haemolytica* local isolate (a) and comparison with the reference ATCC strain (b).

Lkt presence was assessed by the percentage of leukocyte viability at different time intervals of culture. With the Mha local isolate, Lkt secretion started after 7 h of incubation and peaked at 8-9 h. After 10 h of fermentation, the toxin concentration dropped substantially, despite the presence of high bacterial density. With the reference ATCC 43270 strain, cytotoxicity peaked at 7 h and regressed rapidly (Figures-9a and b).

### Pathogenicity in mice

The pathogenicity of the Mha isolate was assessed by inoculating two groups of mice with an infective dose of 10^9^ bacteria/mL. G1 (n=30) was infected with 0.5 mL of supernatant and the supernatant was diluted to 1/5 and 1/10, and G2 (n=30) was inoculated with 0.5 mL of pure fresh bacterial suspensions (1×10^9^ CFU/mL), with 5×10^8^ and 1×10^8^ CFU/mL. In G2, 100% of mice died when inoculated with both the supernatant and culture, and 80% died within 5 min when injected with the supernatant. At the dilutions tested, 40% of mice died in 14 h with the supernatant and 23.2 h with the culture (Table-1). No mortality was observed in mice infected with bacteria washed 3 times.

**Table 1:** Percentage of mortality and time of death in mice infected with *Mannheimia haemolytica* isolate with different concentrations.

Pathogenicity in Mice at 3 different doses				
Bacteria concentration (UFC/mL)	Leukotoxin		Bacteria culture	
	
	Mortality (%)	Lethal time	Mortality (%)	Lethal time
1.10^9^	100	3.3 h	100	18.4 h
5.10^8^	40	9 h	40	23.2 h
1.10^8^	30	13.4 h	0	-

**Figure-10:**
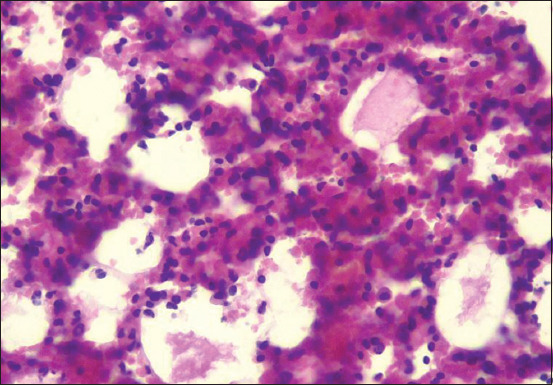
lung: Congestive-edematous alveolitis (hematoxylin and eosin 40×).

In mice infected with Mha, an early stage of bronchopneumonia was observed, characterized by the engorged pulmonary blood vessels of the alveolar septa, with blood (hyperemia) associated with the accumulation of edematous pale pink fluid mixed with few leukocytes in the alveolar space (congestivo-edematous alveolitis) (Figure-10).

### Pathogenicity in sheep and goats

**Figure-11:**
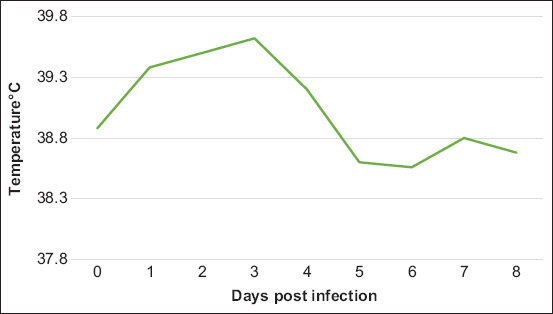
Average rectal temperature of animals infected with MHA1 strain.

Goats and sheep infected with Mha did not show any clinical symptoms; their appetite remained normal, respiratory frequency did not exceed the normal range, and no nasal discharge was observed during the observation period of 22 d. However, all infected animals presented hyperthermia above 39°C for 4day post infection (dpi) with a peak registered on3 dpi (Figure-11).

The three injected goats and one sheep seroconverted as early as 7 dpi and remained positive during the 3 weeks of the experiment. One sheep did not show any antibody response after experimental infection. All sheep and goats were negative for antibodies before inoculation.

**Figure-12:**
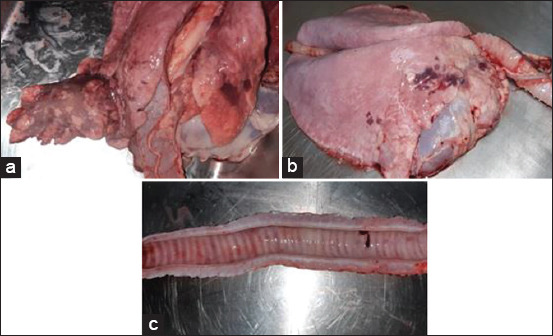
(a-c) Macroscopic characteristic lesions in pneumonic lung.

At the postmortem examination, the lungs of goats and sheep inoculated with Mha showed bronchopneumonia lesions characterized by red to gray-brown consolidated areas of cranial lobes (Figure-12a). The lymph nodes, especially the pulmonary nodes, were congested and hypertrophied. In addition, respiratory airways, including the trachea, were slightly congested (Figures-12b and c).

Mha was detected using PCR in the nasal and oropharyngeal swabs of infected animals from day 3 to 12 pi, with the maximum nasal shedding in sheep [cycle threshold (Ct) 19.9]. At the postmortem examination, the infected sheep showed a Ct of 21.9 in the trachea, Ct 31.4 in lymph nodes, and Ct 38.2 in the lung of one sheep. Goats showed a Ct of 24 in the trachea, Ct 31 in lymph nodes, and Ct 27 in the lung.

### Histopathology

**Figure-13:**
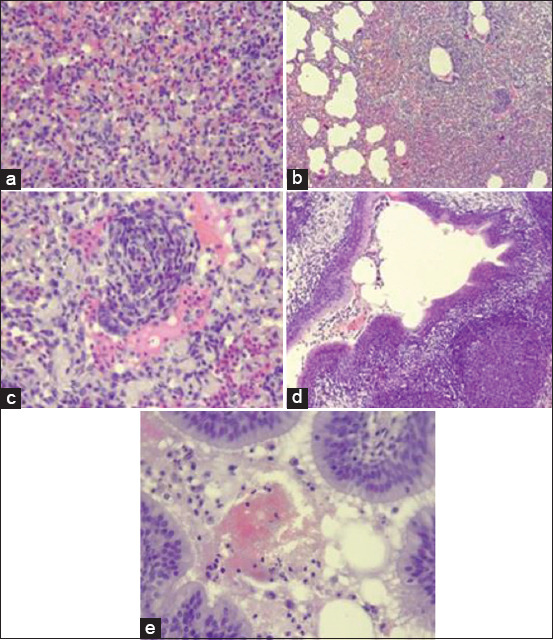
(a-e) Histopathological changes in the pneumonic lungs.

In sheep and goats infected with Mha, fibrinopurulent bronchopneumonia was observed, characterized by leukocytes, predominately neutrophils, fibrinous exudate, and cellular debris (Figure-13a, H&E 40×) in the alveoli lumen. Clusters of inflammatory cells with elongated nuclei (oat cells) are seen (arrow) (Figure-13b, H&E 20×; Figure-13c, H&E 40×). The airways are filled with fibrinopurulent exudate (fibrin plus neutrophils and cellular debris). The bronchiolar epithelium is hyperplasic, the lamina propria is infiltrated with mixed inflammatory cells, and a marked lymphofollicular accumulation (Figure-13d, H&E 40×).

## Discussion

Mha is the major cause of severe pneumonia in respiratory diseases; it represents a real threat to livestock farming development and its contribution to the rural economy. Mha is a commensal organism of the upper respiratory tract and nasopharynx of healthy ruminants; it can colonize the lower respiratory tract, causing pneumonia during stress, such as a viral infection, transportation, or stress associated with weaning, dehorning, and shipping. This bacterium is usually involved in respiratory infections as a primary or secondary pathogen [23].

This study characterized a Mha strain isolated from a dead lamb with acute respiratory signs. Natural infection due to the Mha has been described mainly in cattle [23-25] and rarely in sheep, goats [26,27], or camels [28].

Clinical observations and laboratory investigations revealed that Mha is the causative agent of atypical pneumonia that causes mortality in lambs. Biochemical and molecular analyses by PCR identified this isolate as Mha, serotype A1, the most frequently reported type of pneumonia due to Mha [29]. Detection of the Mha gene *Rpt2* is used commonly for identification, as reported by several authors, resulting in an amplified product of 1022 bp [30,31]. Phylogenetic analyses comparing partial *Rpt2* gene sequences of the Moroccan field isolate with reference and variant strain sequences revealed that the Moroccan isolate was homologous with the American genotype, especially Mha (USDA/ARS/USMARC/184) isolated in the USA in 2013. This homology in genotype distribution among the Mha isolates observed agrees with the findings of Legesse *et al*. [15], which revealed the existence of three genotypes of Mha circulating in the study areas consistent with the site of isolation. The growth kinetics of the Mha isolate in broth media showed a latency phase of 5 h, followed by an exponential phase of 3.5 h and a stationary phase of 1.5 h. The comparative growth kinetics between the local isolate and the reference strain of bovine origin (ATCC) revealed a higher bacterial concentration with the isolate than with the referred ATCC strain with 1 log difference.

Using SDS-PAGE and Western blotting techniques, we detected Lkt in the culture supernatant during the exponential growth phase, 7-9 h of incubation. As reported previously, the intensity of the specific Lkt band, as detected by Western blotting, correlates with bacterial growth kinetics [10].

Leukotoxin secretion was also detected by quantifying neutrophil extracellular DNA; a clear difference was noted between untreated and treated cells with the Mha supernatant of both the isolate and the reference strain. This method did not allow us to differentiate between the two strains regarding the NET. May-Grunwald–Giemsa staining allowed the visualization of cytopathic effects on neutrophils after treatment with the Mha culture supernatant. The observed effect, called neutrophil extracellular DNA, has been reported to be specific to leukotoxin; circulating cell-free DNA is detected as a marker of NETosis in diseases, such as pneumonia. Differentiation between dead and live cells is observed by staining with FDA-PI, as reported by Jones and Senft [20].

Lkt is the main virulence factor of fibronecrotizing pneumonia of cattle and sheep; it was identified as a major component of culture supernatants [6,32]. However, few studies were published on the production and titration of Mha leukotoxin [10,33]. The cultures of the isolate in our report showed significant secretion of specific Lkt, as displayed by leukocyte toxicity in agreement with a previous study [19]. Lkt secretion is linked to the exponential growth phase during culture, as reported by Tucci *et al*. [10]. However, Du Preez *et al*. [32] indicated that Lkt production was onlypartially associated with growth.

In this study, there is linearity between OD, bacterial enumeration, and Lkt secretion. Compared with the reference strain, the local isolate showed more cytotoxicity and virulence in mice. This can be explained by the reduced number of laboratory passages of the isolate.

This study confirms that virulence is linked to the Lkt, as mice died when injected with a sterile supernatant. Mortality occurs rapidly within 3 h at a dose of 10^9^. Low or no mortality was registered with mice injected with the culture or washed bacteria. These results agree with the previous studies that demonstrated that Lkt is the primary virulence factor of Mha [10,34].

Our results and those of Sebbar *et al*. [11] suggest that toxicity studies in mice represent a tool to evaluate immunogenicity using a laboratory animal model rather than goats or sheep. Animals infected with Mha did not show any clinical signs; bacteria alone did not reproduce symptoms in infected animals, and other considerations, such as stress and viral predisposition, are necessary for the disease expression [35]. In the absence of such factors, it can be difficult to induce respiratory diseases in experimental animals. However, all infected animals showed hyperthermia, and at necropsy, they presented various degrees of lung lesions, partially consolidated, with an irregular color. The same histopathological changes were observed in the lung tissues of infected goats, sheep, and mice by Nejiban *et al*. [31]. Therefore, the injected dose (10^9^ CFU) seems to be appropriate to reproduce lesions in animals. The results obtained indicate that Lkt is the primary virulence factor in the pathogenesis of pneumonia in small ruminants. Animals infected with Mha seroconverted in the ELISA antibody test at D7. One sheep did not seroconvert despite the presence of bacteria in tracheal and nasal swabs, probably because of the absence of systemic invasion of Mha or a late antibody response. That was confirmed by a maximum nasal shedding between 3 and 12 dpi. In our experimental conditions, Mha did not cause any clinical symptoms; however, in the field, coinfection with other pathogens might aggravate the disease.

## Conclusion

This study investigated the biological and molecular characterization of the Mha strain isolated from a lamb that died from severe acute pneumonia. Growth kinetics in BHI media has been reported, and the cytotoxins secreted were detected by NETosis and quantified by cytotoxicity tests. The growth of the isolated bacteria had taken 9 to 10 h with an important Lkt secretion compared to the reference strain of Mha. Experimental infection induces mortality in mice due to the Lkt present in the supernatant. Hyperthermia and characteristic lesions in the trachea and lung were noted when the sheep and goat were injected with the supernatant.

## Authors’ Contributions

DB: Performed experiments, isolation, identification, and culture of the isolates; analyzed and drafted the manuscript. NS participated in the identification and culture of the isolates. ZB participated in the revision of the manuscript. NSa carried out staining tests. FZF performed and analysis of data of cytotoxicity titration. ZBa performed the pathogenicity study on mice, goats, and sheep. SF carried out sequencing and phylogenetic analysis. NT performed and analysis of the histopathology study. OFF and KOT participated in the design and the follow-up of the study. ME participated in the design of the study, manuscript drafting and data analysis and interpretation. All authors read and approved the final manuscript.
